# The Composition and the Structure of MCC/Eisosomes in *Neurospora crassa*

**DOI:** 10.3389/fmicb.2020.02115

**Published:** 2020-09-18

**Authors:** Qin Yang, Frank Kempken

**Affiliations:** Department of Genetics and Molecular Biology, Botanical Institute and Botanic Garden, Kiel University, Kiel, Germany

**Keywords:** eisosome, *Neurospora crassa*, mass spectrometry, eisosomal proteins, cellular localization

## Abstract

MCC/eisosomes are protein-organized domains in the plasma membrane of fungi and algae. However, the composition and function(s) of MCC/eisosomes in the filamentous fungus *Neurospora crassa* were previously unknown. To identify proteins that localize to MCC/eisosomes in *N. crassa*, we isolated proteins that co-purified with the core MCC/eisosome protein LSP-1, which was tagged with GFP. Proteins that co-fractionated with LSP-1:GFP were then identified by mass spectrometry. Eighteen proteins were GFP-tagged and used to identify six proteins that highly colocalized with the MCC/eisosome marker LSP-1:RFP, while five other proteins showed partial overlap with MCC/eisosomes. Seven of these proteins showed amino acid sequence homology with proteins known to localize to MCC/eisosomes in the yeast *Saccharomyces cerevisiae*. However, homologs of three proteins known to localize to MCC/eisosomes in *S. cerevisiae* (Can1, Pkh1/2, and Fhn1) were not found to colocalize with MCC/eisosome proteins in *N. crassa* by fluorescence microscopy. Interestingly, one new eisosome protein (glutamine-fructose-6-phosphate aminotransferase, gene ID: NCU07366) was detected in our studies. These findings demonstrate that there are interspecies differences of the protein composition of MCC/eisosomes. To gain further insight, molecular modeling and bioinformatics analysis of the identified proteins were used to propose the organization of MCC/eisosomes in *N. crassa*. A model will be discussed for how the broad range of functions predicted for the proteins localized to MCC/eisosomes, including cell wall synthesis, response and signaling, transmembrane transport, and actin organization, suggests that MCC/eisosomes act as organizing centers in the plasma membrane.

## Introduction

The biological membrane system is complex and has several critical functions for biochemical processes in living cells. It segments into coexisting compartments, which enable spatiotemporal segregation and coordination of different activities within the system (Zahumensky and Malinsky, 2019). For example, it has been recognized for a long time that there are heterogeneous lateral compartments in the plasma membrane of all cells (Léon and Teis, 2018). In fungi, plasma membrane lateral microdomains have been reported. These microdomains are named by specific marker proteins: the membrane compartment that contains Can1 (MCC), the membrane compartment that contains Pma1 (MCP), and the membrane compartment that contains Torc2 (target of rapamycin complex 2) (MCT) (Karotki et al., 2011; Kolláth-Leiβ and Kempken, 2017). Eisosomes are protein complexes in fungi and algae, which are localized at the plasma membrane, associated with the MCC domain (Douglas and Konopka, 2014). Eisosomes were initially proposed in the yeast *S. cerevisiae* in 2006 (Walther et al., 2006). Currently eisosomes are described as large cytosolic protein scaffolds that stabilize the invaginations of the integral membrane portion MCC (Zahumensky and Malinsky, 2019). Eisosomes have furrow shapes and consist of distinct protein compositions (Babst, 2019). However, the exact composition of eisosomes remains controversial, even in *S. cerevisiae*, where eisosomes have been most studied (Walther et al., 2006). Pil1, Lsp1, and Sur7 are the first reported MCC/eisosomal components, and eisosomes are reported to be composed primarily of Pil1 and Lsp1 in *S. cerevisiae* (Walther et al., 2006). Pil1 and Lsp1 both contain a BAR (Bin/Amphiphysin/Rvs) and could bind the plasma membrane and promote the formation of curvatures (Olivera-Couto et al., 2011; Ziółkowska et al., 2011; Bharat et al., 2018). In yeast, Pkh1 and Pkh2 regulate eisosome assembly and organization via the Pkh signaling pathway (Walther et al., 2007). Slm1 and Slm2 are another pair of eisosomal proteins found in *S. cerevisiae.* These proteins have a BAR domain and act in actin cytoskeleton organization (Grossmann et al., 2008; Olivera-Couto et al., 2011; Douglas and Konopka, 2014). Furthermore, some proteins with unknown functions have been discovered to localize with eisosomes, including Seg1 and Ygr130c (Deng et al., 2009), Eis1 and Mdg1 (Grossmann et al., 2008; Aguilar et al., 2010), and Msc3 (Moreira et al., 2009; Douglas et al., 2011b).

For other fungi, only a few eisosomal proteins have been discovered so far. The eisosome compositions appear to be different in the analyzed fungi (Scazzocchio et al., 2011). In *Aspergillus nidulans*, PilA, PilB, and SurG were studied and described as core components of MCC/eisosomes (Athanasopoulos et al., 2013). Among them, PilA and PilB are homologs of the *S. cerevisiae* eisosomal proteins Pil1/Lsp1. SurG is a homolog of the MCC protein Sur7 in yeast (Athanasopoulos et al., 2013). The three MCC/eisosomal proteins in *A. nidulans* exhibit different characteristics between different types of cells, for example, PilA and SurG are necessary for MCC/eisosome organization in conidia, whereas in germlings of ascospores, the punctate structures are composed only of PilA, PilB is diffused in the cytoplasm, and SurG is localized in vacuoles and endosomes (Vangelatos et al., 2010; Athanasopoulos et al., 2013). In addition, an Nce102 homolog was reported to be another MCC protein in *A. nidulans* (Athanasopoulos et al., 2015).

Not only the composition but also the formation and function(s) of eisosomes remain unclear. The spatial location of the eisosome is regulated, as the restricted distribution found in *Neurospora crassa* was not only observed in the unicellular fungus *S. pombe* and *S. cerevisiae* (Kabeche et al., 2011; Douglas and Konopka, 2014) but also discovered in the filamentous fungi *A. nidulans* (Athanasopoulos et al., 2013). What is more, eisosomes are stably distributed at the cell periphery (Kabeche et al., 2011; Athanasopoulos et al., 2015; Lacy et al., 2017) and are always separated from each other; however, the mechanism for that is still unknown (Moreira et al., 2009; Douglas and Konopka, 2014). The functions of MCC/eisosomes have been studied in *S. cerevisiae* (Olivera-Couto and Aguilar, 2012; Bartlett et al., 2015), *Candida albicans* (Alvarez et al., 2009; Foderaro et al., 2017), *Beauveria bassiana* (Zhang et al., 2017), *Ashbya gossypii* (Seger et al., 2011), and *A. nidulans* (Vangelatos et al., 2010; Athanasopoulos et al., 2015). MCC/Eisosomes have been reported to be involved in diverse cellular processes, such as cellular signaling (Olivera-Couto and Aguilar, 2012), membrane domain formation (Bartlett et al., 2015), polarized growth (Seger et al., 2011), cell wall synthesis and morphogenesis (Douglas and Konopka, 2014; Foderaro et al., 2017), and pathogen virulence (Zhang et al., 2017). Even so, the significant function(s) of MCC/eisosomes is still poorly understood. In *S. cerevisiae* no fitness defects were detected in cells lacking eisosomes (Moseley, 2018), and in *A. nidulans* deletion of the core eisosomal proteins does not lead to any obvious growth defects and does not affect viability or germination of ascospores (Vangelatos et al., 2010; Athanasopoulos et al., 2013).

*Neurospora crassa* is a well-established model organism for genetics, biochemistry, and molecular biology since it was first described by Payen in 1843 (Roche et al., 2014). The genome is about 40 megabases containing 10,000 protein-coding genes (Galagan et al., 2003). Recently, the LSP-1 protein in *N. crassa* was detected as a functional and core component of the eisosome (Yang et al. Eisosomes Show Different Features in Morphologically Identical Hyphae Germinating from Sexual and Asexual Spores in *Neurospora crassa*.). Nevertheless, the composition of the MCC/eisosome is still poorly understood in *N. crassa*. Nor has the function(s) of MCC/eisosomes been reported in *N. crassa*. As a morphologically complex multicellular fungus, *N. crassa* has sexual and asexual life cycles with a range of cell types, which make it much more complicated than the unicellular yeast *Saccharomyces* (Seale, 1973; Bistis et al., 2003). Besides, in *N. crassa* there are many characteristic biological activities including hyphal fusion occurring at different stages (Fleißner et al., 2008), the establishment and maintenance of hyphal polarity (Riquelme et al., 2011), and so on, which are not found in unicellular yeasts. These make *N. crassa* a better-suited model system to deeply study the composition and function(s) of MCC/eisosomes. Our data reveal the MCC/eisosome composition in *N. crassa* and bring new insights into the MCC/eisosome function(s).

## Results

### Isolation and Enrichment of Eisosomal Proteins

**FIGURE 1:**
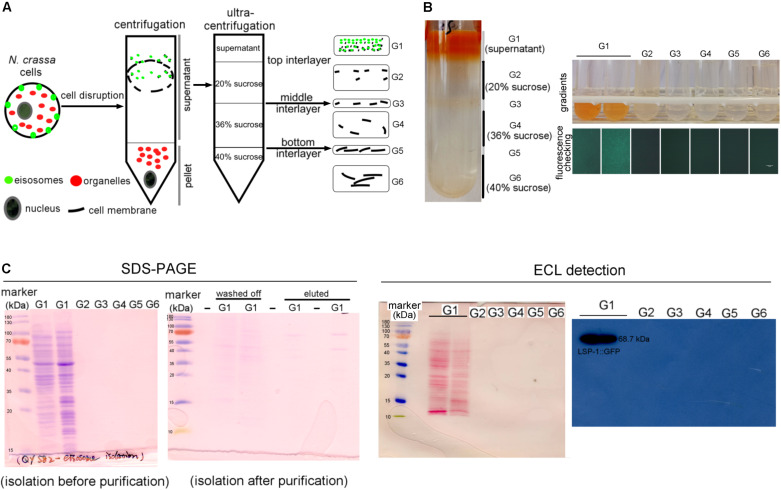
Isolation of eisosomes and the western blot result. **(A)** The schematic diagram of the eisosome isolation process. Eisosomes were discovered in the supernatant after the normal centrifugation. G1–G6 present the six gradients separated after the ultracentrifugation. **(B)** The result of the eisosome isolation after the ultracentrifugation. The six fractions were transferred into six small tubes and checked under a fluorescence microscope. It seems there were two layers in the G1 fragment, so we transferred them into two tubes and found they were the same during later examinations. As the fluorescence result shown here, LSP-1:GFP was specifically detected in gradient G1. **(C)** The SDS-PAGE images of the primary isolation and the enriched isolation, which reflects most of the unspecific proteins, were removed after the purification. The ECL detection results reveal that proteins were successfully transferred on a nitrocellulose membrane and specific bands of LSP-1:GFP (68.7 kDa) were detected from G1 sample without any other bands. There was no band detected from G2–G6 on films during ECL detection.

To isolate eisosomes, the core eisosomal protein LSP-1 (Yang et al. Eisosomes Show Different Features in Morphologically Identical Hyphae Germinating from Sexual and Asexual Spores in *Neurospora crassa*.) was fused with green fluorescent protein (GFP) and used as a reporter. *N. crassa* cells were ground to powder in liquid nitrogen and put into solution, centrifuged twice at different speeds to remove nuclei, organelles, and intact cells in the pellet. During the isolation process, we determined that the eisosomes were in the supernatants after centrifugation by checking the GFP fluorescence with a fluorescence microscope (Figure 1A). The supernatant was then loaded onto a 40–36–20% sucrose gradient for ultracentrifugation (Figures 1A,B). After the ultracentrifugation six gradient fractions were taken, and the eisosomes were specifically detected in the topmost fraction (Figure 1B). Therefore, the first fraction was taken for further anti-GFP immunoprecipitation purification using magnetic beads. As LSP-1 is the fundamental and core component of eisosomes, the eluted fraction after the immunoprecipitation magnetic separation was enriched for eisosomal proteins. An isolation from a WT strain that expresses no GFP fused proteins was isolated and purified in the same way from the beginning and acted as a blank control in the subsequent mass spectrometry analysis, because it contains proteins non-specifically bound to the anti-GFP immunoprecipitation magnetic beads.

### Western Blot Analysis

LSP-1:GFP was confirmed in the isolation by western blot (Figure 1C). The signal of the fusion protein was significantly strong using enhanced chemiluminescence (ECL) detection. The band exists at around 70 kDa, which is consistent of the size of LSP-1:GFP (68.7 kDa) in theory (Figure 1C). The eisosome fragments were successfully extracted in the isolation. In addition, the by-product proteins, which are not combined with LSP-1, were washed away during the enrichment process (Figure 1C). The SDS PAGE result showed that there were many proteins of different sizes isolated after the pull-down enrichment. The protein concentration decreased approximately fivefold after the enrichment. An anti-GFP antibody was used for the western blot. All the samples used for further study in our research were detected by fluorescence analysis and showed strong GFP signals before western blotting.

### Identification of Proteins From the Enriched Eisosomal Protein Fragment

**FIGURE 2:**
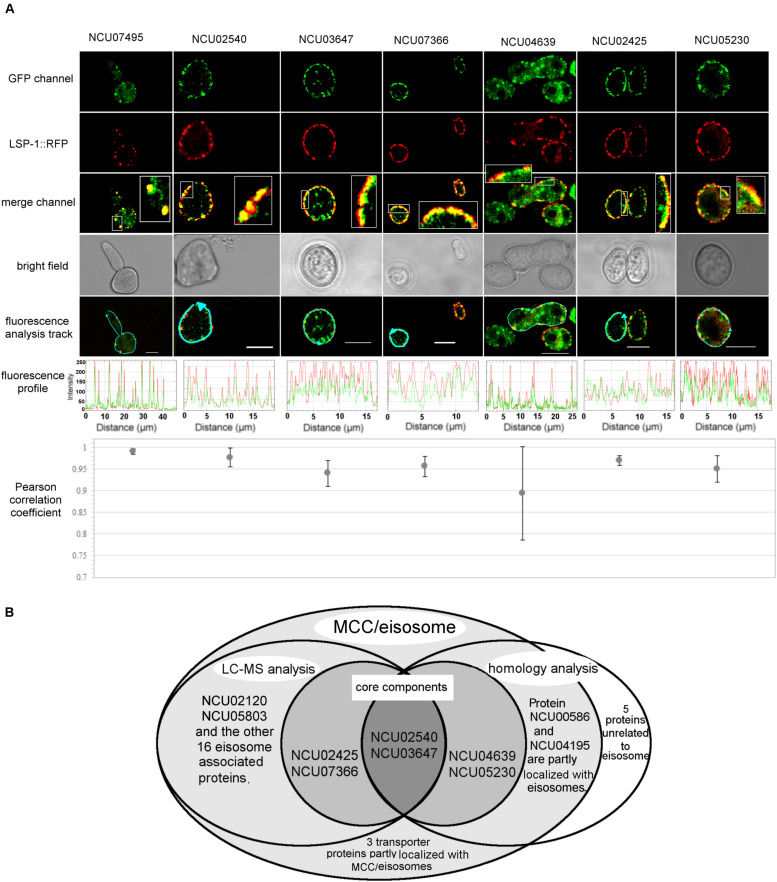
Localization verification of the core MCC/eisosomal proteins identified in our study. **(A)** The dual fluorescence colocalization analysis of LSP-1:RFP and MCC/eisosomal proteins tagged by GFP. The GFP and RFP signals merged well, which reveals that the MCC/eisosomal proteins are highly colocalized with MCC/eisosomes. The fluorescence profiles and the Pearson correlation coefficients confirmed the colocalization between the MCC/eisosomal proteins identified in the current study and MCC/eisosomes. The arrow lines in the fluorescence analysis track channel shows the routes of the fluorescence density profile analysis. The boxes are magnifications to show more details. The Pearson correlation coefficient value is between –1 and + 1, where the value closer to 1 means more positive correlation; 0 means no correlation; the value closer to –1 means more negative correlation. Mean ± SD, n ≥ three experiments, statistical analysis using one-way analysis of variance (*P* = 0.17, means those protein equally colocalized with MCC/eisosomes). Scale bar = 5 μm. **(B)** Overview of the LC-MS analysis, homolog analysis and transporter protein detection for MCC/eisosomal protein identification.

Once it was confirmed that the enriched fractions contained the LSP-1:GFP eisosomal protein, liquid chromatography-tandem mass spectrometry (LC-MS) was used to identify proteins in the purified sample. As the ultra-centrifugation could easily extract proteins whose specific gravities are similar to eisosomes and the unspecific proteins may not be completely removed after GFP pull-down, it was necessary to set up control groups. The enriched sample was analyzed as the experiment group, while the isolation without GFP pull-down was set as the conditional control, and the enriched sample from WT was used as the blank control. The non-specifically isolated proteins, which were not combined with LSP-1, were largely removed by the specific immune pull-down, whereas the eisosomal proteins were mainly retained (Figure 1C). In this way, the ratio of eisosomal components was greatly increased in the enriched sample. Considering that there might be proteins apart from eisosomes non-specifically adhering to the magnet beads during the pull-down process, the peptide hits existing in the LC-MS blank control list will all be deleted from the final eisosomal protein list despite the augmentation of their ratio. With this condition, in the comparison of the experimental group and the conditional control, we detected 22 (excluding LSP-1) eisosomal proteins from the LC-MS analysis (Table 1). All of the 22 proteins were detected from the top hits with a protein threshold of 95%, peptide threshold of 95%, normalized total spectra bigger than 25, and more than five peptide contigs were detected in the LC-MS analysis. Additionally, the ratio of these proteins in the purification sample were at least five-fold greater than those in the conditional control. In our study, we ranked the 22 top hits (excluding LSP-1) by their normalized total spectral parameter and assumed that the proteins with normalized spectra higher than 70 are core eisosomal proteins in *N. crass* (Table 1). In this way, we discovered six (excluding LSP-1) fundamental eisosomal proteins from the LC-MS analysis. We subsequently tested our hypothesis by dual fluorescence localization analysis. Four of the assumed core eisosomal proteins were successfully tagged by green fluorescence protein (GFP) and individually expressed with the eisosomal reporter protein LSP-1:RFP (red fluorescence protein). We examined the colocalization of each protein with the known core eisosomal protein LSP-1 and finally confirmed that these four proteins are all core eisosomal components (Figure 2). The dual fluorescence intensity profiles visually showed the overlaps of the signals of GFP and RFP (Figure 2A). Even more intriguing, the four proteins all got extremely high Pearson’s correlation coefficient values in the colocalization analysis with LSP-1, which confidently indicates that they are core components of eisosomes in *N. crassa* cells (Figure 2A). The eisosomal protein LSP-1 was chosen as the positive control (Pearson’s *r* = 0.99 ± 0.01) and one of the non-eisosome protein (NCU03571) was chosen as a negative control (Pearson’s *r* = 0.015 ± 0.02) for the colocalization analysis (Supplementary Figure S1). Based on the controls, we define the proteins with their Pearson’s correlation coefficients larger than 0.6 to be eisosome correlated proteins; the proteins with Pearson’s correlation coefficients smaller than 0.2 to be eisosome unrelated proteins; and the proteins with Pearson’s correlation coefficients between 0.2 and 0.6 to be partly correlated to eisosomes.

**TABLE 1:** LC-MS identification result*.

Gene ID**	Normalized spectra		Ratio	Protein coverage	Relationship to eisosomes
(FungiDB)	Control	Purification	(Control/Purified)		
NCU07495	12	611	50.9	66%	highly colocalized
NCU03647	3	232	77.3	47%	highly colocalized
NCU02540	12	138	11.5	54%	highly colocalized
NCU02120	0	108	−	31%	related
NCU02425	1	73	73.00	30%	highly colocalized
NCU05803	6	71	11.83	20%	related
NCU07366	10	71	7.10	37%	highly colocalized
NCU03897	12	67	5.58	27%	related
NCU06943	12	60	5.00	37%	related
NCU02839	8	53	6.63	41%	related
NCU06544	3	51	17.00	23%	related
NCU09808	6	48	8.00	34%	related
NCU08875	4	44	11.00	20%	related
NCU08699	6	41	6.830	40%	related
NCU02207	8	41	5.13	47%	related
NCU05887	2	37	18.50	34%	related
NCU09700	4	33	8.25	32%	related
NCU08340	3	31	10.33	57%	related
NCU03102	4	28	7.00	31%	related
NCU00410	2	26	13.00	29%	related
NCU07567	3	26	8.67	28%	related
NCU09119	3	26	8.67	30%	related
NCU08920	5	25	5.00	26%	related

*Protein threshold = 95%; peptide threshold = 95%; peptides contigs ≥ 5. **The gene annotation and production are listed in Supplementary Table S1.

### Homolog Analysis of MCC/Eisosomal Proteins

Some MCC/eisosomal proteins have been discovered since 2006 (Walther et al., 2006). Most of these are found in *S. cerevisiae* and a few homologs of the yeast MCC/eisosomal proteins have been found in other fungi. Using bioinformatic methods, we found 11 homologs (excluding LSP-1) of MCC/eisosomal proteins in *N. crassa* (Table 2). Most of these matched MCC/eisosomal proteins in *S. cerevisiae*, which are all well-known MCC/eisosomal proteins but were never confirmed as eisosome components in *N. crassa*. However, it is surprising that the predicted MCC/eisosomal protein list obtained by homolog analysis hardly coincided with the LC-MS eisosomal protein list. Except for LSP-1, there were only two proteins (NCU02540 and NCU03647) that existed on both lists (Figure 2B). These two proteins have been verified as core components of eisosomes in *N. crassa*, as was described above. Thus there are 20 proteins newly discovered to be associated with eisosomes (four core eisosomal proteins and 16 candidate eisosomal proteins) from the eisosome enrichment and LC-MS analysis in this study.

**TABLE 2:** Putative MCC/eisosomal proteins from homolog analysis.

Gene and production (Gene ID from fungiDB)	GO terms (from fungiDB)	Relationship to MCC/eisosomes	Homolog in yeast
NCU07495 sphingolipid long chain base-responsive protein LSP1	eisosome; response to heat	highly colocalized	Pil1/Lsp1
NCU02540 meiotic expression up-regulated protein 14	response to light stimulus	highly colocalized	Pil1/Lsp1
NCU03647 hypothetical protein	cellular response to oxidative stress	highly colocalized	Ykl050c
NCU04639 non-classical export protein (Nce102)	membrane-associating domain; membrane	highly colocalized	Nce102
NCU05230 hypothetical protein	membrane-associating domain	highly colocalized	Nce102
NCU04195 purine-cytosine permease FCY21	cellular component; membrane	partly colocalized	Lyp1
NCU00586 non-anchored cell wall protein-6	cellular component; fungal-type cell wall	partly colocalized	Sur7
NCU03571 serine/threonine protein kinase	protein phosphorylation; response to oxidative stress	unrelated	Pkh1
NCU05198 general amino acid permease	cellular component; membrane	unrelated	Lyp1/Can1
NCU07334 uracil permease	cellular component; membrane	unrelated	Can1
NCU07754 methionine permease	cellular component; membrane	unrelated	Lyp1
NCU04809 MFS phospholipid transporter	integral component of membrane	unrelated	Fhn1
**Total**	12		

For the other nine putative MCC/eisosomal proteins from the homolog prediction, which did not correspond with the LC-MS identification results, more experiment evidence was needed to validate whether they were truly MCC/eisosomal proteins. We performed fluorescence trace analysis to identify their localization with MCC/eisosomes. Each of these proteins was fused with GFP and expressed individually with LSP-1:RFP. We detected two proteins (NCU04639, a homolog of Nce102; NCU05230 hypothetical protein, another homolog of Nce102) to be core MCC components (Pearson’s *r* = 0.89 ± 0.11 and 0.95 ± 0.03) (Table 2 and Figures 2A,B); two proteins partly locating with MCC/eisosomes (NCU00586, Pearson’s *r* = 0.32 ± 0.01; NCU04195, Pearson’s *r* = 0.23 ± 0.04) (Table 2 and Figure 2B and Supplementary Figures S2A,B); four proteins not locating with MCC/eisosomes at all (Pearson correlation coefficients all smaller than 0.1) (Table 2 and Supplementary Figures S2C,D); and there was one protein that showed no fluorescence signal (NCU04809, Table 2). For every fluorescence colocalization experiment, we performed a fluorescence density profile analysis, calculated the Pearson correlation coefficient, and did statistical analysis to calculate the colocalization relationship between these proteins and MCC/eisosomes separately (Figure 2A). The use of Pearson correlation coefficients was based on the metrics of a positive control versus a negative control, which is described at the end of section “Identification of Proteins From the Enriched Eisosomal Protein Fragment.” In *N. crassa*, NCU04639 is the non-classical export protein (Nce102) and is a homolog of the yeast MCC core component Nce102. In our research, NCU04639 was determined to be a core MCC component at the cortex of *N. crassa* cells (Figure 2A). The fluorescence density profile experiment showed that the GFP signals from NCU04639 and the RFP signals from eisosomes significantly overlap. The value of the Person’s coefficient is 0.89 ± 0.11 (Figure 2A). NCU05230 is a hypothetical protein that is homologous to the yeast MCC protein Nce102. NCU05230 was not among the final LC-MS identified protein list (it was detected in the eisosome enrichment LC-MS experiment, but was eliminated by the filter parameters). The fluorescence microscopy showed that NCU05230 was a core component of MCC at the *N. crassa* cell membrane (Figure 2A). The dual fluorescence signals from NCU05230 and LSP-1 perfectly coincide in the fluorescence density profiles. The Pearson correlation coefficient value was 0.95 ± 0.03 (Figure 2A). These strongly indicate that NCU04639 and NCU05230 are core MCC components in *N. crassa*. With regard to the two proteins partly localized at MCC/eisosomes, we determined that NCU00586 is the homolog of the yeast Sur7 protein and NCU04195 is the homolog of LYP1 in *S. cerevisiae*. These two proteins have multifarious distributions in *N. crassa* cells. The fluorescence analysis captures showed that they form spot patterns at the cell membrane and cytoplasmic pools as well as spot patterns in the cytoplasm (Supplementary Figure 2A). The fluorescence density profile analysis showed that the GFP signals from these two proteins are not completely overlapped with the fluorescence from LSP-1:RFP (Supplementary Figure 2A). At most of the LSP-1:RFP fluorescence profile peaks, there were co-locating GFP fluorescence profile peaks for those proteins; nevertheless, the area and the value of those peaks did not perfectly coincide with each other as the core eisosomal components’ did. The Pearson’s coefficient values for NCU00586 and NCU04195 were 0.32 ± 0.01, 0.23 ± 0.04, respectively (Supplementary Figure 2B), which demonstrates that NCU00586 and NCU04195 were partly localized with MCC/eisosomes but were not among the core components of MCC/eisosomes in *N. crassa*. These proteins may dynamically localize at MCC/eisosomes for their function(s) in different biological processes under different growth conditions, and dissociate from MCC/eisosomes after that.

In our research, five MCC/eisosome homologous proteins were determined not to be components of MCC/eisosomes (Figure 2B and Supplementary Figures S2C,D). It was determined that the *N. crassa* proteins NCU03571, NCU05198, NCU07334, and NCU07754 are homologs of MCC/eisosomal proteins Pkh1, Can1/Lyp1, Can1, and Lyp1, respectively. Surprisingly, in our study, we found that these four proteins were not components of MCC/eisosomes in *N. crassa*. Their GFP signals did not overlap with the LSP-1:RFP signals in the fluorescence density profiles (Supplementary Figure S2C). As for NCU04809, it is a homolog of MCC component Fhn1 in *S. cerevisiae.* We did not detect any significant fluorescence signals at the cell membrane from the GFP fused protein in the transformed cell.

### Three Transporter Proteins Were Detected to Partly Localize at MCC/Eisosomes

In addition to the MCC/eisosomal proteins discovered from the LC-MS and the homolog analysis, we detected three transporters that partly localize at MCC/eisosomes in *N. crassa* cells (Table 3 and Figure 2B). It is well known that MCC/eisosomes and transporter proteins are all localized at the cell membrane (Douglas et al., 2011a; Coronas-Serna et al., 2018). In order to determine whether there are connections between them, we fused GFP to some transporter proteins that are transmembrane transporters and have sequence similarities with the yeast MCC/eisosomal proteins, and performed the dual fluorescence localization analysis. NCU06352 (oligopeptide transporter 4), NCU06384 (MFS sugar transporter), and NCU01065 (ammonium transporter MEP2) were discovered to associate with MCC/eisosomes (Table 3 and Supplementary Figures S2A,B). Their colocalization statistical analysis yielded Pearson correlation coefficients of 0.33 ± 0.10, 0.35 ± 0.04, and 0.34 ± 0.08, respectively (Table 3 and Supplementary Figure S2B), which indicates that they all associate with MCC/eisosomes in *N. crassa* but not strongly. This relationship suggests that MCC/eisosomes may participate in transmembrane transport in *N. crassa* cells.

**TABLE 3:** Three transporter proteins partly localized at MCC/eisosomes.

Gene and production (Gene ID from fungiDB)	Function prediction (GO terms from funfiDB)	Protein length	Relationship to MCC/eisosomes
NCU06352 oligopeptide transporter 4	transmembrane transport	1057 aa	partly colocalized
NCU01065 ammonium transporter MEP2	cellular component; membrane	481 aa	partly colocalized
NCU06384 MFS sugar transporter	integral component of membrane	532 aa	partly colocalized
Total	3		

### Characteristics of the Core MCC/Eisosomal Proteins

**FIGURE 3:**
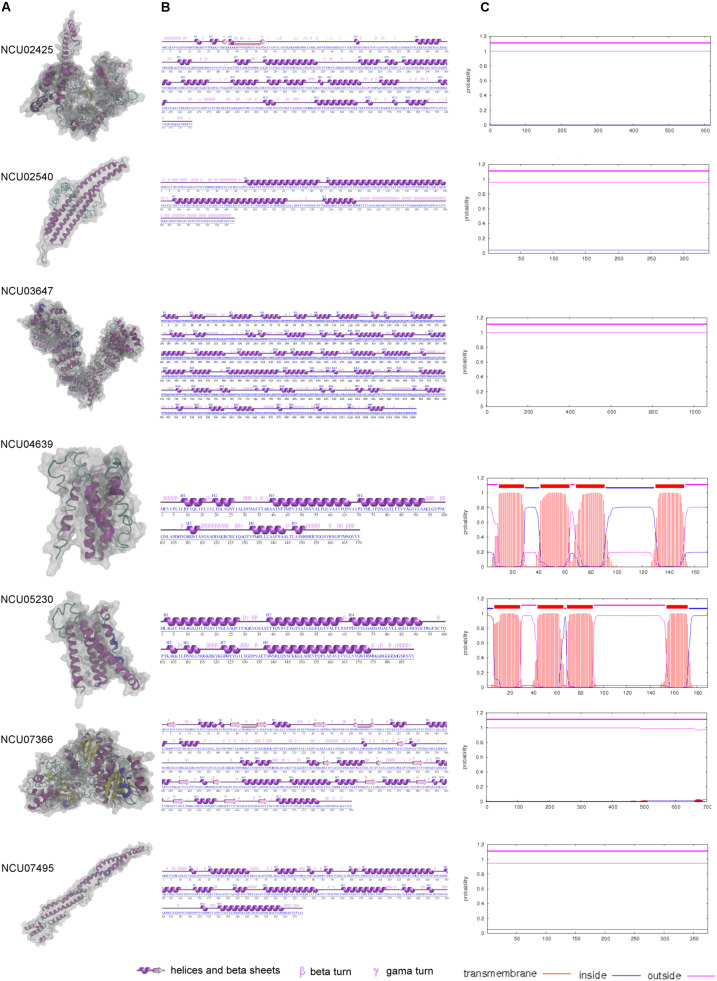
The modeling and characteristic analysis of MCC/eisosomal core components. **(A)** The column of the protein structures modeled with I-Tasser and visualized with VMD. **(B)** The secondary structure characteristics of each core components. **(C)** The transmembrane analysis of each core MCC/eisosomal protein.

The six core MCC/eisosomal proteins identified in our study, as well as the LSP-1 protein, were analyzed with the I-Tasser and ProFunc approaches for their characteristics. The predicted protein structures are shown in Figure 3. Confidence scores (C-scores) to estimate the quality of predicted structures were given after the predictions. The score is in a range of [−5,2]: higher scores reflect models of better quality. Models with C-scores > −1.5 generally have correct fold (Roy et al., 2010).

For the core eisosomal component LSP-1, the model (C-score = −1.19) was generated by using the templates, including 3pltA, in the PDB library without any restraints. LSP-1 contains a conserved domain that belongs to the Pil1 superfamily. The model has a filament shape (Figure 3A) consisting of 374 residues. It contains 16 α-helices accounting for 58.3% of the residues. The rest of the residues (41.7%) include other secondary structures such as 3_10_-helix, beta turns, and gamma turns, and there is no β-sheet in the model (Figure 3B).

The protein NCU02540 had a high similarity to LSP-1. The model (C-score = −2.44) also has a filament shape (Figure 3A) and was mainly generated by using the template 3pltA in the PDB library. NCU02540 has the same Pil1 superfamily conserved domain as LSP-1. In contrast to LSP-1, the NCU02540 model has only four α-helices accounting for 53.7% of the 339 residues and the helices individually consist of more than 18 residues. In the model, the remaining 46.3% of the residues are composed of other secondary structures such as beta turns and gamma turns. There is no β-sheet in the model (Figure 3B).

The model (C-score = −0.32) of NCU03647 was constructed according to the threading template alignment including 5yz0A in PDB. It has 1062 residues with 69 helices including 52 α-helices accounting for 51.5% and 17 3_10_-helices accounting for 4.1%. In addition, there are two β-sheets accounting for 0.3%, with 44.1% consisting of other secondary structures such as β-bulges, helix-helix interactions, beta, and gamma turns (Figure 3B). The model has a spiral shape (Figure 3A).

The X-ray crystallographic structure of glucosamine-6-P synthase 2j6hA and some other structures in the PDB library served as templates without any restrains to build the model (C-score = −0.21) of protein NCU07366, which is a new eisosomal protein identified in our study. The model has 22 helices including 18 α-helices accounting for 30.4% and five 3_10_-helices taking up 2.3% (with the last α-helices and 3_10_-helices blending into one helix) of the total 700 residues. There are 17 β-sheets, which accounts for 12.1% of the residues in the model, and 55.1% of other secondary structures (Figure 3B). The model has an approximately dumbbell shape (Figure 3A).

Based on templates from the PDB entry such as 6aayA, the protein structure prediction was performed. The model (C-score = −0.96) of NCU02425 has a hooked shape (Figure 3A) and contains 20 α-helices and four 3_10_-helices accounting for 42.4% of the total 616 residues; two short β-sheets accounting for 1% and other secondary structures accounting for 56.7% (Figure 3B).

NCU04639 is a core MCC protein in the homolog analysis but was not among fragments obtained in the LSP-1 pull-down procedure. It was modeled according to the threading template alignment of X-ray real structures including the 5tjvA protein in the PDB library. The predicted model (C-score = −2.93) has a barrel-like shape (Figure 3A) with seven α-helices (including three long helices consisting of 13, 24, and 29 residues and three short helices composed of five, five, eight, and nine residues) accounting for 54.7% of 170 residues. There is no β-sheet in the model and 45.3% of other secondary structures including helix-helix interactions, beta, and gamma turns (Figure 3B). It is a transmembrane protein localized at MCC (Figure 3C).

NCU05230 is the other coreMCC protein identified from homolog analysis but missing from in the final LC-MS result. The model (C-score = −2.38) was generated by using known structural proteins including 4amjA and 5tjvA in the PDB library as templates. The length and shape of the model is similar to NCU04639 (Figure 3A). It was determined to be another transmembrane protein localized at MCC (Figure 3C). The secondary structure of the NCU05230 model consists of seven α-helices accounting for 68.3% (four long α-helices, respectively, composed of 24, 26, 27, and 38 residues; three short ones with four, four, and six residues) of 189 residues, and two 3_10_-helices accounting for 2.1%. There is no β-sheet and the other secondary structures in the model are helix-helix interactions, beta, and gamma turns accounting for 29.6% (Figure 3B).

### Structure of Eisosomes

**FIGURE 4:**
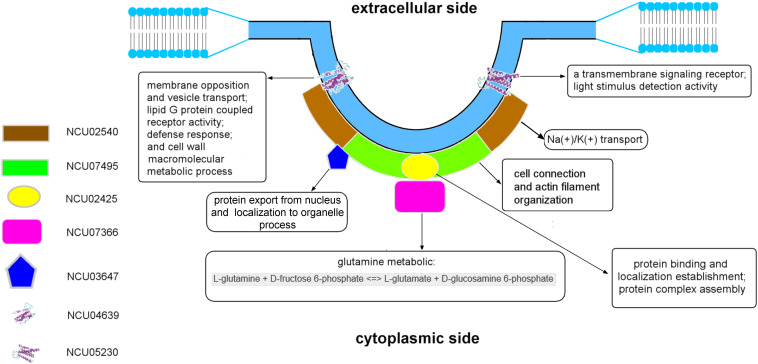
The model of the eisosome structure in *N. crassa*.

The MCC/eisosomal core components from LC-MS and homolog analysis were analyzed by TMHMM server to detect their transmembrane location (Figure 3C, probabilities are shown on the figure). Interestingly, the four proteins that combine with LSP-1, which were identified in the LC-MS analysis, show no transmembrane domains. They are all localized outside of the phospholipid bilayer. In contrast, NCU04639 and NCU05230, which were identified from the homolog analysis, rather than in the LSP-1 pull-down isolation, are both transmembrane proteins. This is consistent with the protein modeling result. Each of the transmembrane proteins has four transmembrane regions. The transmembrane parts of NCU04639 are between residues 10–29, 42–64, 69–91, and 130–152 while the transmembrane areas of NCU05230 lay between residues 7–29, 44–66, 69–91, and 154–172. The beginning of protein NCU04639 is out of the membrane lipid bilayers (probability = 0.81) while protein NCU05230 starts from the inside of the cell membrane (probability = 0.97). The second and third transmembrane areas in both proteins are of same length and the locations are similar/same. The first and last transmembrane regions of NCU04639 are three and four residues longer than NCU05230. All the above considered, in the model of the eisosome, it is clear that LSP-1 and the proteins combined with it are localized on the cytosolic side of the cell membrane. They constitute the framework of the furrow structure of eisosomes while NCU04639 and NCU05230 traverse the cell membrane and are localized at the furrow (MCC) on the cell membrane (Figure 4). These differences could account for the fact that NCU04639 and NCU05230 are both MCC core components that can be identified by homolog analysis but cannot be enriched by the LSP-1 pull-down procedure. The MCC/eisosomal proteins that partly associate with MCC/eisosomes may periodically be temporarily colocalized at MCC/eisosomes as they perform their own dynamic and special function(s).

## Discussion

In our study, we took two approaches to identify MCC/eisosome proteins in *N. crassa*. Firstly, we isolated the eisosomal proteins complexed to LSP-1 in *N. crassa* (Figure 1). The fluorescence from attached GFP conveniently and precisely reported the presence or absence of eisosomes during the isolation and enrichment. The proteins co-fractionated with LSP-1:GFP were then identified with LC-MS analysis (Table 1). In addition, we examined homologs of proteins known to localize to MCC/eisosomes in *S. cerevisiae* (Table 2). These approaches resulted in identification of six proteins that were highly localized to MCC/eisosomes and five others that showed partial overlap with MCC/eisosomes (Figure 2 and Supplementary Figures S2A,B).

As the results of the co-fractionation, 22 proteins were identified by mass spectrometry (Table 1). Six of them were selected as prime eisosomal protein candidates with high confidence filter parameters (described in the see section “Results”). We successfully tagged four of the prime eisosomal protein candidates while the other two proteins (NCU02120, coding a 5540 bp gene, and NCU05803, coding an 8293 bp gene) were not successfully amplified in the cloning experiment due to a technical problem. The four GFP tagged proteins were verified to be highly localized to MCC/eisosomes (Figure 2), and two of them were only identified in the LC-MS analysis but not in the homolog analysis: NCU02425 has a region of amino acid similarity with Slm1/2 in yeast, and NCU07366 is a new eisosome core component detected in our study. For the proteins NCU02120 and NCU05803, they are extremely likely to be eisosome localized proteins because their identification parameter is higher than some of the verified highly-localized-eisosomal proteins (Table 1). Nevertheless, we cautiously consider them as eisosome-associated proteins in our study until they are confirmed in further research. Interestingly, we found two proteins (NCU04639 and NCU05230) from homolog analysis highly colocalized with MCC/eisosomes but not co-fractionated with LSP1:GFP during the isolation (Figure 2). This suggests that MCC/eisosomes are not a structure consisting of only one protein cluster, but rather a complex membrane domain including protein clusters, independent protein components, and phospholipids.

As the results for the analysis of the homologs of *S. cerevisiae* MCC/eisosome proteins, 11 proteins (excluding LSP-1) were successfully GFP tagged (Table 2). Four of them highly localized to MCC/eisosomes; two of them partial localized to MCC/eisosomes; and five of them did not localize to MCC/eisosomes (Figure 2 and Supplementary Figure S2). One possible explanation for these results may be due to the growth conditions during the research, as MCC/eisosomes have dynamic compositions, which are depending on different growth conditions (Babst, 2019; Appadurai et al., 2020). Our results indicate that the composition of MCC/eisosomes in *N. crassa* is different from that in *S. cerevisiae.* Apparently MCC/eisosomes’ compositions and assembly are dissimilar in different species, which is consistent with published data (Kabeche et al., 2011; Scazzocchio et al., 2011; Douglas and Konopka, 2014). For example, Sur7 and Slm1 were detected as two MCC/eisosomal proteins in budding yeast (Walther et al., 2006; Malinsky and Opekarová, 2016; Busto et al., 2018), and the Sur7 homolog was even examined as a core MCC component, whereas the homologs of Slm1 and Sur7 were not part of MCC/eisosomes in fission yeast (Kabeche et al., 2011). Likewise, in our study using *N. crassa*, the Sur7 homolog (Table 2) was found to be partly localized at MCC/eisosomes only (Supplementary Figures S2A,B). Furthermore, the homologs of the *S. cerevisiae* MCC/eisosomal components Can1, Pkh1/2, Lyp1, and Fhn1 (Olivera-Couto and Aguilar, 2012) were not found to be localized in MCC/eisosomes in *N. crassa* (Figure 2 and Supplementary Figure S2). As already pointed out, this may be due to the growth conditions during the research.

Regarding the results of the molecular modeling and bioinformatics (Figures 3, 4 and Supplementary Results), the eisosome component NCU03647 has functional annotations in nuclear protein transfer (Cscores^*GO*^ = 0.22, GO-score = 0.43); NCU07366 has annotations of function in the glucosamine-6-phosphate (GlcN6P) synthesis process (Cscore^*EC*^ = 0.61); NCU04639 has a conserved domain associated with vesicle transport. It raises the possibility MCC/eisosomes may have function(s) in protein modification and secretion. Furthermore, GlcN6P is an essential precursor of cell wall components chitin (Bulik et al., 2003) and NCU04639 also has functional annotations in the cell wall macromolecule metabolic process (GO-score = 0.44), which indicates that MCC/eisosomes, consistent with previous data, may be required for proper cell wall morphogenesis including Pil1, Lsp1, Sur7, and Slm1/2 (Alvarez et al., 2008; Wang et al., 2011, 2016; Douglas et al., 2013; Douglas and Konopka, 2019). Additionally, NCU05230 and NCU04639 both have a transmembrane receptor functional annotation and the former is related to light stimulus detection (GO-score = 0.56) while the latter is associated with defense response (Cscores^*GO*^ = 0.22, GO-score = 0.35). Therefore, MCC/eisosomes may have function(s) in signaling pathways in *N. crassa* cells. Eisosomes may also be involved with cation transport and membrane potential, because NCU02540 is predicted to be an ion pump at the cell membrane (Cscore^*EC*^ = 0.117). As for LSP-1, it has an actin filament organization domain (GO-score = 0.36), which suggests that eisosomes most probably have cytoskeleton related function(s) at the plasma membrane. Last but not least, eisosome components NCU02425 and LSP-1 may be required to establish the location of eisosomes on the plasma membrane.

The eisosomal model of eisosomes in Figure 4 is based on the results of modeling and bioinformatics. Proteins with functional annotations regarding the assembly and localization of MCC/eisosomes were arranged to the plasma membrane, including NCU02425, NCU02540, and NCU07495; other eisosomal components were recruited at different positions of eisosomes according to their annotations on cellular processes (see Supplementary Results). According to the molecular modeling and bioinformatic analysis of the identified MCC/eisosomal proteins (Figures 3, 4 and Supplementary Results), some eisosomal proteins were found to have annotations of functions on the assembly and localization of eisosomes. Interestingly other eisosomal components were found to have functional annotations on cellular processes, such as cell wall synthesis, response and signaling, transmembrane transport, and actin organization. These data will inspire future experiments in the field, especially to elucidate the functions and structures of eisosomes.

The structural and functional model for *N. crassa* MCC/eisosomes presented in this study provide an important intermediate step in the ongoing functional studies on the roles of eisosomes, that will further define the unique properties of these specialized membrane domains.

## Materials and Methods

### Media and Growth Conditions

The media and growth conditions in our study were as described previously (Kollath-Leiß et al., 2014).

### Strains

The *N. crassa* strains used in our study are listed in Supplementary Table S2. The strains constructed in our study are based on the histidine auxotrophic strains FGSC #6103 and 9716 (Fungal Genetics Stock Center; Kansas City, MO, United States). The genes fused with *gfp/rfp* integrate into *N. crass* chromosomes by homologous recombination (Margolin et al., 1997).

The bacterial strain *E. coli* XL1-Blue [*recA1, endA1, gyrA96, thi-1, hsdR17, supE44, relA1, lac F’proAB lacIqZ*Δ*M15 Tn10 (Tetr)*] (200249, Stratagene) was used for electroporation and chemical transformations. A *ccdB* survival *E. coli* strain [*F-mcrA*Δ*(mrr-hsdRMS-mcrBC)*Φ*80lacZ*Δ*M15*Δ*lacX74 recA1 ara*Δ*139*Δ*(ara-leu)7697 galU galK rpsL (StrR) endA1 nupG fhuA:IS2*] (A10460, Invitrogen) was used for Gateway cloning.

### Eisosome Fragment Isolation

The conidia of the LSP-1:GFP strain were harvested and soaked in sterile water for 3 h. Samples were centrifuged (Allegra X-30, Beckman Coulter) at 4000 rpm for 5 min and then the supernatants were removed. The fresh pellets were collected, and the harvested conidia were weighed. As soon as possible after weighing, the conidia were frozen in liquid nitrogen for 5 min and then stored at −80°C overnight. Samples of the frozen conidia were next ground into a fine powder in liquid nitrogen as quickly as possible using mortars and pestles. According to their fresh weight, the powders were immediately dissolved into the appropriate amount of solution buffer (5 ml/g). The dissolved samples were then centrifuged at 1000 × *g* for 10 min at 4°C (Avanti J-20 XP, Beckman Coulter), then the supernatants were collected and centrifuged again at 11000 × *g* for 20 min at 4°C (Avanti J-20 XP, Beckman Coulter). The second supernatants were collected and put on ice. The supernatants and pellets were checked after every centrifugation using a fluorescence microscope (ECLIPSE Ci system plus INTENSILIGHT C-HGFI 130w lamp, Nikon) to make sure that the eisosome/LSP-1:GFP complexes were successfully isolated.

The gradients for ultracentrifugation were set as follows: 40% sucrose 10 ml, 36% sucrose 10 ml, and 20% sucrose 12 ml. The gradients were transferred to the centrifugation tubes from the bottom (40% sucrose) to the top (20% sucrose) in a cool room and stored at 4°C for 2 h. Then a maximum of 2 ml of the final supernatants were quickly loaded onto the cold gradient in the cool room. Next the tubes containing the samples and gradients were placed into centrifugation cups and centrifuged in an ultracentrifuge TST28.38 rotor at 24000 rpm for 1 h at 4°C (Optima L-90K Ultracentrifuge, Beckman Coulter). After the ultracentrifugation, each fragment was carefully collected from each tube as soon as possible. The fluorescence of each fragment was checked with a fluorescence microscope (ECLIPSE Ci system plus INTENSILIGHT C-HGFI 130w lamp, Nikon) (a 40 × /NA0.75 [plan fluor] objective lens was used to acquire images) and then they were stored at −80°C.

### Enrichment of the Isolated Fragments

The isolation of eisosome fragment was enriched by using the μMACS GFP Isolation Kit (130-091-125, Miltenyi Biotec). Fifty μl of the GFP-Tag Microbeads was added from the kit to 1 ml of each protein sample. They were mixed well and were keep on ice for 30 min. The micro-columns in the kit were put on the magnet rack and then were equilibrated with 200 μl of lysis buffer. After that the protein-microbeads mixes were transferred into the equilibrated micro-columns. Next the micro-columns containing protein samples were washed four times with a total of 800 μl of washing buffer one from the kit before washing them once with 100 μl of washing buffer 2 from the kit. The elution buffer from the kit was heated to 95°C initially. First, 20 μl of the pre-heated elution buffer was added into each micro-column and they were incubated at room temperature for 5 min. Finally the LSP-1:GFP combined proteins were eluted with 50 μl of the 95°C elution buffer.

### Western Blot

The protein samples were prepared and loaded onto a 5% stacking gel and 15% separating gel. After the samples were resolved by SDS-PAGE, they were transferred onto a 0.1 μm nitrocellulose transfer membrane (G161476, Whatman) under 3.5 mA/cm (Léon and Teis, 2018) (E835, Consort) for 30 min at room temperature. After that, the proteins were probed with an HPR conjugated mouse anti-GFP antibody (working dilution: 1:5000) (SIGMA-ALDRICH). Signals were detected by ECL detection.

### LC-MS Analysis

The isolated and enriched proteins were measured by the Bradford method and sent to the Max Planck Institute of Molecular Cell Biology and Genetics (Dresden, Germany) for LC-MS procedure. Peptide sequences were searched against the *N. crassa* database in FungiDB to try to match the detected spectra and identify the protein using Scaffold 4 software (Proteome Software). Strict parameters were set (protein threshold >95%, peptide threshold >95% with at least five peptide hits) to obtain proteins identified with high confidence. We set as controls proteins identified from the wild type strain as well as from the unpurified LSP-1:GFP isolation, and compared these with the enriched fragments to identify the eisosomal proteins.

### Homolog Analysis of MCC/Eisosomal Proteins

Eisosomes were initially reported and have been best studied in *S. cerevisiae*. Until now LSP-1 is the only confidently identified eisosomal protein in *N. crassa*. As two eukaryotic model organisms, both fungi have sequenced genomics. From the *Saccharomyces* Genome Database (SGD) we obtained the DNA and protein sequences of MCC/eisosome components in *S. cerevisiae*. By blasting against the *N. crassa* database, some MCC/eisosomal protein homologs were detected. Other bioinformatic methods such as gene ontology (GO) analysis were also performed to identify more MCC/eisosomal protein homologs in *N. crassa*. Most of the homolog proteins have conserved domains of *S. cerevisiae* MCC/eisosomal proteins.

### Molecular Experiments in the Study

DNA isolation and DNA amplification were performed as described previously (Mercker et al., 2009; Kollath-Leiß et al., 2014).

Gel electrophoresis was normally performed on 1% agarose at 20 V for 20 min and then the voltage was increased to 100–120 V for 1 h (Mercker et al., 2009).

A NucleoSpin Gel and PCR Clean-up kit (740609.50, Macherey-Nagel) was used for gel elution.

DNA restrictions were processed using endonuclease enzymes from New England BioLabs, following the manufacturer’s recommendations.

USER enzyme (M5505S, New England BioLabs), CloneJET PCR cloning Kit (K1231, Thermo scientific), and T4 ligase (M0202S, New England BioLabs) were employed for cloning experiments.

### Plasmid Construction

The plasmids used in our study are listed in Supplementary Table S3. They were constructed as described previously (Kollath-Leiß et al., 2014).

Plasmids pQY867 and pQY868 carry the *N. crassa lsp-1* promoter which controlled the expression of the *N. crassa lsp-1:gfp* sequence and *N. crassa lsp-1:rfp* sequence, respectively.

The other plasmids individually carry *gfp* tagged genes coding LSP-1 and putative MCC/eisosomal proteins detected by the LC-MS procedure and homolog analysis. The *cfp* promoter is a strong promoter from *N. crassa* and was used to control the expression of the potential MCC/eisosomal genes fused with *egfp/trfp* in these plasmids (Temporini et al., 2004). These putative MCC/eisosomal genes in the LC-MS and homolog analysis lists were separately amplified using the oligos listed in Supplementary Table S4. The *egfp/trfp* fragment was amplified as previously described (Mercker et al., 2009; Kollath-Leiß et al., 2014).

### Electroporation and Selection of Transformants

Electroporation of *N. crassa* conidia was carried out as described previously (Kumar et al., 2013). The induction and selection of homozygous single colonies were performed in the same way as described previously (Kollath-Leiß et al., 2014). All cloning and transformation experiments were conducted in accordance with the requirements of the German gene technology law (GenTG).

### Construction of Dual Fluorescence Strains

*Neurospora crassa* strains of the same mating type, which individually contain GFP and RFP, were cultivated in VMM + S slants at 25°C for 7 days to get enough macroconidia. The spores of each strain were harvested and washed with 1 M sorbitol. Then they were counted under a microscope, and their final concentrations were made to 6.5 × 10^7^ spores/ml. One hundred μl of each conidial sample from the GFP strains were individually mixed with the same amount of conidia from the LSP-1:RFP strain. They were incubated at 25°C for 30 min and then were pipetted into VMM + S slants. The slants were incubated at 25°C in light for 5 to 6 days. During the time the spores of both fluorescence were germinating, fusing, and developing into strains with dual fluorescence proteins. The conidial spores from each dual fluorescence strain were inoculated onto VMM + S thin agar plates and were cultivated briefly or were directly checked for fluorescence under the fluorescence microscope (ECLIPSE Ci system plus INTENSILIGHT C-HGFI 130w lamp, Nikon).

### Microscopy

Fungi of different genotypes were incubated on thin agar plates before microscopy.

Microscope analysis was performed with a fluorescence microscope (ECLIPSE Ci system plus INTENSILIGHT C-HGFI 130 w lamp, Nikon). 40 × /NA0.75 (plan fluor), 100 × /NA1.30 oil (plan fluor) objective lenses were used to acquire images. Immersion oil (Type N, Nikon) was used with the oil objective lens for observation and image acquisition. Epi-Fluorescence filter G-2E/C (TRITC), EX 540/25, DM 565, BA 605/55 was used for red fluorescence. A GigE camera (DFK 23U274, Imaging Source) was used to capture photos. The acquisition software was NIS elements D basic (Nikon). Images were captured at room temperature (22–25°C).

Confocal fluorescence analysis was performed using a confocal laser scanning microscope (Leica, TCS SP5) and images were captured with the Leica LAS AF Lite Software. GFP was excited with 488 nm light and emission was detected at 500–550 nm, while RFP was excited with 543 nm light and emission was detected at 590–610 nm.

During each microscope experiment, including CLSM, a blank control of WT was set to make sure there was no auto fluorescence at the experimental settings.

### Fluorescence and Colocalization Analysis

The analysis of the fluorescence and colocalization of LSP-1 and putative MCC/eisosomal proteins was performed by ImageJ software after microscopy. The fluorescence intensity profiles of GFP and RFP at the cell membrane were determined to display the degree of overlay of these dual fluorescence, which visually indicated the localization relationship of LSP-1 and other proteins. Pearson’s coefficients were calculated to indicate the statistical significance of the colocalization frequency.

### Gateway Cloning

In our study, we modified the Gateway method and created a one-step destination vector cloning system, which is a convenient high throughput system for *N. crassa* expression studies. First, the *N. crassa* expression vector was equipped with the *attP*-flanked DNA fragment (Supplementary Figure S3), then inserted the *egfp* ORF behind the *attP*-flanked DNA fragment. The modified vector was used as a donor as well as an entry vector during the one-step Gateway cloning and is shown in Supplementary Figure S3. It contains a fragment of 1618 base pairs of *his-3* sequence for the homologous recombination induced chromosomal integrations (Margolin et al., 1997). The *cfp* promoter and *arg* terminator also controlled the expression of genes between them (Mercker et al., 2009). The *attP_1_/attP_2_* flanks were necessary for the recombination reaction between the PCR product and the vector. Between the *attP_1_/attP_2_* flanks are the chloramphenicol fragment and *ccdB* fragment for colony selection, which would all be replaced by the target genes after the cloning. The *attB*_1_ and *attB*_2_ linker were added in front of the forward and reverse primers of the target genes. The PCR was performed with the high fidelity *Pwo*-DNA-Polymerase (732–3262, PeqLab). The accurately amplified fragments were directly mixed with the modified vector and the BP reactions were performed using the Gateway BP Clonase (11789020, Thermo Fisher Scientific) to generate the destination plasmids. The transformation and colony selections were described above. The destination plasmids were verified by DNA sequencing.

### Protein Modeling and Prediction

We used I-TASSER and ProFunc servers for protein modeling, and structural and functional analysis in our study. The transmembrane prediction was performed with TMHMM2.0.

## Data Availability Statement

All datasets presented in this study are included in the article/Supplementary Material. The mass spectrometry proteomics data have been deposited to the ProteomeXchange Consortium via the PRIDE (Perez-Riverol et al., 2019) partner repository with the dataset identifier PXD020724.

## Author Contributions

FK conceived the study and participated in data analysis and discussion. QY carried out the experiments, analyzed the data, and drafted the manuscript. Both authors contributed to the article and approved the submitted version.

## Conflict of Interest

The authors declare that the research was conducted in the absence of any commercial or financial relationships that could be construed as a potential conflict of interest.

## References

[B1] Aguilar P. S. Fröhlich F. Rehman M. Shales M. Ulitsky I. Olivera-Couto A. (2010). A plasma-membrane E-MAP reveals links of the eisosome with sphingolipid metabolism and endosomal trafficking. *Nat. Struct. Mol. Biol.* 17 901–908. 10.1038/nsmb.1829 20526336 PMC3073498

[B2] Alvarez F. J. Douglas L. M. Konopka J. B. (2009). The Sur7 protein resides in punctate membrane subdomains and mediates spatial regulation of cell wall synthesis in *Candida albicans*. *Commun. Integr. Biol.* 2 76–77. 10.1091/mbc.E08-05-0479.76 19704893 PMC2686348

[B3] Alvarez F. J. Douglas L. M. Rosebrock A. Konopka J. B. (2008). The Sur7 protein regulates plasma membrane organization and prevents intracellular cell wall growth in *Candida albicans*. *Mol. Biol. Cell* 19 5214–5225. 10.1091/mbc.e08-05-0479 18799621 PMC2592640

[B4] Appadurai D. Gay L. Moharir A. Lang M. J. Duncan M. C. Schmidt O. (2020). Plasma membrane tension regulates eisosome structure and function. *Mol. Biol. Cell* 4 287–303. 10.1091/mbc.E19-04-0218 31851579 PMC7183764

[B5] Athanasopoulos A. Boleti H. Scazzocchio C. Sophianopoulou V. (2013). Eisosome distribution and localization in the meiotic progeny of *Aspergillus nidulans*. *Fungal Genet. Biol.* 53 84–96. 10.1016/j.fgb.2013.01.002 23395641

[B6] Athanasopoulos A. Gournas C. Amillis S. Sophianopoulou V. (2015). Characterization of AnNce102 and its role in eisosome stability and sphingolipid biosynthesis. *Sci. Rep.* 5:15200. 10.1038/srep15200 26468899 PMC4606592

[B7] Babst M. (2019). Eisosomes at the intersection of TORC1 and TORC2 regulation. *Traffic* 20 543–551. 10.1111/tra.12651 31038844 PMC6663646

[B8] Bartlett K. Gadila S. K. G. Tenay B. Mcdermott H. Alcox B. Kim K. (2015). TORC2 and eisosomes are spatially interdependent, requiring optimal level of phosphatidylinositol 4, 5-bisphosphate for their integrity. *J. Biosci.* 40 299–311. 10.1007/s12038-015-9526-9524 25963258

[B9] Bharat T. A. M. Hoffmann P. C. Kukulski W. (2018). Correlative microscopy of vitreous sections provides insights into BAR-domain organization in situ. *Structure* 26 879–886. 10.1016/j.str.2018.03.015 29681471 PMC5992340

[B10] Bistis G. N. Perkins D. D. Read N. D. (2003). Different cell types in *Neurospora crassa*. *Fungal Genet. Rep.* 50 17–19. 10.4148/1941-4765.1154

[B11] Bulik D. A. Olczak M. Lucero H. A. Osmond B. C. Robbins P. W. Specht C. A. (2003). Chitin synthesis in *Saccharomyces cerevisiae* in response to supplementation of growth medium with glucosamine and cell wall stress. *Eukaryot. Cell* 2 886–900. 10.1128/EC.2.5.886-900.2003 14555471 PMC219353

[B12] Busto J. V. Elting A. Haase D. Spira F. Kuhlman J. Schäfer-Herte M. (2018). Lateral plasma membrane compartmentalization links protein function and turnover. *EMBO J.* 37:e99473. 10.15252/embj.201899473 29976762 PMC6092676

[B13] Coronas-Serna J. M. Fernández-Acero T. Molina M. Cid V. J. (2018). A humanized yeast-based toolkit for monitoring phosphatidylinositol 3-kinase activity at both single cell and population levels. *Microb. Cell* 5 545–554. 10.15698/mic2018.12.660 30533419 PMC6282018

[B14] Deng C. Xiong X. Krutchinsky A. N. (2009). Unifying fluorescence microscopy and mass spectrometry for studying protein complexes in cells. *Mol. Cell. Proteomics* 8 1413–1423. 10.1074/mcp.M800397-MCP200 19269952 PMC2690482

[B15] Douglas L. M. Konopka J. B. (2014). Fungal membrane organization: the eisosome concept. *Annu. Rev. Microbiol.* 68 377–393. 10.1146/annurev-micro-091313-103507 25002088

[B16] Douglas L. M. Konopka J. B. (2019). Plasma membrane architecture protects *Candida albicans* from killing by copper. *PLoS Genet.* 15:e1007911. 10.1371/journal.pgen.1007911 30633741 PMC6345494

[B17] Douglas L. M. Wang H. X. Keppler-Ross S. Dean N. Konopka J. B. (2011a). Sur7 promotes plasma membrane organization and is needed for resistance to stressful conditions and to the invasive growth and virulence of *Candida albicans*. *mBio* 3:e00254-11 10.1128/mBio.00254-211 PMC3244266 22202230

[B18] Douglas L. M. Wang H. X. Li L. Konopka J. B. (2011b). Membrane Compartment Occupied by Can1 (MCC) and Eisosome Subdomains of the Fungal Plasma Membrane. *Membranes* 1 394–411. 10.3390/membranes1040394 22368779 PMC3285718

[B19] Douglas L. M. Wang H. X. Konopka J. B. (2013). The MARVEL domain protein Nce102 regulates actin organization and invasive growth of Candida. *mBio* 4:e00723-13 10.1128/mBio.00723-713 PMC3870249 24281718

[B20] Fleißner A. Simonin A. R. Glass N. L. (2008). Cell fusion in the filamentous fungus, *Neurospora crassa*. *Methods Mol. Biol.* 475 21–38. 10.1007/978-1-59745-250-2_2 18979236

[B21] Foderaro J. E. Douglas L. M. Konopka J. B. (2017). MCC/eisosomes regulate cell wall synthesis and stress responses in fungi. *J. Fungi* 1 1–18. 10.3390/jof3040061 29371577 PMC5753163

[B22] Galagan J. E. Calvo S. E. Borkovich K. A. Selker E. U. Read N. O. Jaffe D. (2003). The genome sequence of the filamentous fungus *Neurospora crassa*. *Nature* 422 859–868. 10.1038/nature01554 12712197

[B23] Grossmann G. Malinsky J. Stahlschmidt W. Loibl M. Weig-Meckl I. Frommer W. B. (2008). Plasma membrane microdomains regulate turnover of transport proteins in yeast. *J. Cell Biol.* 183 1075–1088. 10.1083/jcb.200806035 19064668 PMC2600745

[B24] Kabeche R. Baldissard S. Hammond J. Howard L. Moseley J. B. (2011). The filament-forming protein Pil1 assembles linear eisosomes in fission yeast. *Mol. Biol. Cell* 22 4059–4067. 10.1091/mbc.E11-07-0605 21900489 PMC3204068

[B25] Karotki L. Huiskonen J. T. Stefan C. J. Ziółkowska N. E. Roth R. Surma M. A. (2011). Eisosome proteins assemble into a membrane scaffold. *J. Cell Biol.* 195 889–902. 10.1083/jcb.201104040 22123866 PMC3257569

[B26] Kollath-Leiß K. Bönniger C. Sardar P. Kempken F. (2014). BEM46 shows eisosomal localization and association with tryptophan-derived auxin pathway in *Neurospora crassa*. *Eukaryot. Cell* 13 1051–1063. 10.1128/EC.00061-14 24928924 PMC4135797

[B27] Kolláth-Leiβ K. Kempken F. (2017). “The fungal MCC/eisosome complex: an unfolding story,” in *The Mycota XV*, eds Anke T. Schüffler A. (Berlin: Springer International Publishing AG), 119–130. 10.1007/978-3-319-71740-1_4

[B28] Kumar A. Kollath-Leiß K. Kempken F. (2013). Characterization of bud emergence 46 (BEM46) protein: sequence, structural, phylogenetic and subcellular localization analyses. *Biochem. Biophys. Res. Commun.* 438 526–532. 10.1016/j.bbrc.2013.07.103 23916612

[B29] Lacy M. M. Baddeley D. Berro J. (2017). Single-molecule imaging of the BAR-domain protein Pil1p reveals filament-end dynamics. *Mol. Biol. Cell* 28 2251–2259. 10.1091/mbc.e17-04-0238 28659415 PMC5555653

[B30] Léon S. Teis D. (2018). Functional patchworking at the plasma membrane. *EMBO J.* 37 2–4. 10.15252/embj.2018100144 30061314 PMC6092615

[B31] Malinsky J. Opekarová M. (2016). New insight into the roles of membrane microdomains in physiological activities of fungal cells. *Int. Rev. Cell Mol. Biol.* 325 119–180. 10.1016/bs.ircmb.2016.02.005 27241220

[B32] Margolin B. S. Freitag M. Selker E. U. (1997). Improved plasmids for gene targeting at the his-3 locus of Neurospora crassa by electroporation. *Fungal Genet. Rep.* 44 34–36. 10.4148/1941-4765.1281

[B33] Mercker M. Kollath-Leiß K. Allgaier S. Weiland N. Kempken F. (2009). The BEM46-like protein appears to be essential for hyphal development upon ascospore germination in Neurospora crassa and is targeted to the endoplasmic reticulum. *Curr. Genet.* 55 151–161. 10.1007/s00294-009-0232-233 19238386

[B34] Moreira K. E. Walther T. C. Aguilar P. S. Walter P. (2009). Pil1 controls eisosome biogenesis. *Mol. Biol. Cell* 20 809–818. 10.1091/mbc.E08 19037108 PMC2633383

[B35] Moseley J. B. (2018). Eisosomes. *Curr. Biol.* 28 R376–R378. 10.1016/j.cub.2017.11.073 29689217 PMC6214159

[B36] Olivera-Couto A. Aguilar P. S. (2012). Eisosomes and plasma membrane organization. *Mol. Genet. Genomics* 287 607–620. 10.1007/s00438-012-0706-708 22797686

[B37] Olivera-Couto A. Grana M. Harispe L. Aguilar P. S. (2011). The eisosome core is composed of BAR domain proteins. *Mol. Biol. Cell* 22 2360–2372. 10.1091/mbc.E10-12-1021 21593205 PMC3128537

[B53] Perez-Riverol Y. Csordas A. Bai J. Bernal-Llinares M. Hewapathirana S. Kundu D. J. (2019). The PRIDE database and related tools and resources in 2019: improving support for quantification data. *Nucleic Acids Res.* 47, D442–D450. 10.1093/nar/gky1106 30395289 PMC6323896

[B38] Riquelme M. Yarden O. Bartnicki-Garcia S. Bowman B. Castro-Longoria E. Free S. J. (2011). Architecture and development of the *Neurospora crassa* hypha - a model cell for polarized growth. *Fungal Biol.* 115 446–474. 10.1016/j.funbio.2011.02.008 21640311

[B39] Roche C. M. Loros J. J. McCluskey K. Glass N. L. (2014). *Neurospora crassa*: looking back and looking forward at a model microbe. *Am. J. Bot.* 101 2022–2035. 10.3732/ajb.1400377 25480699

[B40] Roy A. Kucukural A. Zhang Y. (2010). I-TASSER: a unified platform for automated protein structure and function prediction. *Nat. Protoc.* 5 725–738. 10.1038/nprot.2010.5 20360767 PMC2849174

[B41] Scazzocchio C. Vangelatos I. Sophianopoulou V. (2011). Eisosomes and membrane compartments in the ascomycetes: a view from *Aspergillus nidulans*. *Commun. Integr. Biol.* 4 64–68. 10.4161/cib.4.1.13764 21509182 PMC3073274

[B42] Seale T. (1973). Life cycle of *Neurospora crassa* viewed by scanning electron microscopy. *J. Bacteriol.* 113 1015–1025. 10.1128/jb.113.2.1015-1025.1973 4266170 PMC285320

[B43] Seger S. Rischatsch R. Philippsen P. (2011). Formation and stability of eisosomes in the filamentous fungus *Ashbya gossypii*. *J. Cell Sci.* 124 1629–1634. 10.1242/jcs.082487 21525038

[B44] Temporini E. D. Alvarez M. E. Mautino M. R. Folco H. D. Rosa A. L. (2004). The *Neurospora crassa* cfp promoter drives a carbon source-dependent expression of transgenes in filamentous fungi. *J. Appl. Microbiol.* 96 1256–1264. 10.1111/j.1365-2672.2004.02249 15139917

[B45] Vangelatos I. Roumelioti K. Gournas C. Suarez T. Scazzocchio C. Sophianopoulou V. (2010). Eisosome organization in the filamentous ascomycete *Aspergillus nidulans*. *Eukaryot. Cell* 9 1441–1454. 10.1128/EC.00087-10 20693301 PMC2950425

[B46] Walther T. C. Aguilar P. S. Fröhlich F. Chu F. Moreira K. Burlingame A. L. (2007). Pkh-kinases control eisosome assembly and organization. *EMBO J.* 26 4946–4955. 10.1038/sj.emboj.7601933 18034155 PMC2094096

[B47] Walther T. C. Brickner J. H. Aguilar P. S. Bernales S. Walter P. (2006). Eisosomes mark static sites of endocytosis. *Nature* 439 998–1003. 10.1038/Nature04472 16496001

[B48] Wang H. X. Douglas L. M. Aimanianda V. Latgé J.-P. Konopka J. B. (2011). The *Candida albicans* Sur7 protein is needed for proper synthesis of the fibrillar component of the cell wall that confers strength. *Eukaryot. Cell* 10 72–80. 10.1128/EC.00167-110 21115741 PMC3019807

[B49] Wang H. X. Douglas L. M. Veselá P. Rachel R. Malinsky J. Konopka J. B. (2016). Eisosomes promote the ability of Sur7 to regulate plasma membrane organization in *Candida albicans*. *Mol. Biol. Cell* 27 1663–1675. 10.1091/mbc.E16-01-0065 27009204 PMC4865322

[B50] Zahumensky J. Malinsky J. (2019). Role of MCC/Eisosome in Fungal Lipid Homeostasis. *Biomolecules* 9:305. 10.3390/biom9080305 31349700 PMC6723945

[B51] Zhang L. B. Tang L. Ying S. H. Feng M. G. (2017). Two eisosome proteins play opposite roles in autophagic control and sustain cell integrity, function and pathogenicity in *Beauveria bassiana*. *Environ. Microbiol.* 19 2037–2052. 10.1111/1462-2920.13727 28276124

[B52] Ziółkowska N. E. Karotki L. Rehman M. Huiskonen J. T. Walther T. C. (2011). Eisosome-driven plasma membrane organization is mediated by BAR domains. *Nat. Struct. Mol. Biol.* 18 854–856. 10.1038/nsmb.2080 21685922

