# Supplementary material for: The Composition and the Structure of MCC/Eisosomes in Neurospora crassa
Source: Front Microbiol. 2020 Sep 18;11:2115. doi: 10.3389/fmicb.2020.02115 (PMC7533531; doi:10.3389/fmicb.2020.02115)
Supplement: Supplementary file 1 [file Data_Sheet_1.pdf]

Supplemental materials

Structural and functional analysis of the core MCC/eisosomal proteins

Initial checks of the mutant of each of the six core MCC/eisosomal proteins were performed, but no obvious growth phenotype was observed, nor did the mutant of the LSP-1 protein. In order to get a comprehensive overview of the functions of MCC/eisosomes in a shorter timeframe and to inspire future studies on the roles of MCC/eisosomes, a bioinformatic analysis based on the sequence-to-structure-to-function paradigm was performed to predict their structural and functional characteristics. TM-scores (template modeling scores) indicate the topological similarity of protein structures. The TM-score is in a range of (0, 1], higher scores indicating better structural matches. TM-scores > 0.5 indicate that the proteins have similar folds and that the analogs can be used for determining the structure class of the predicted protein (Roy et al., 2010). $Cscores^{GO}$ (confidence scores of gene ontology prediction) and $Cscore^{EC}$ (confidence score of enzyme commission) respectively indicate the strength of predictions of functional annotations and enzyme commissions. These scores both have their range between [0, 1]: higher values indicate more reliable predictions. Additionally, GO-scores are defined as the average weight of the GO terms, where the weights are assigned based on $Cscores^{GO}$. With a GO-score cutoff = 0.5, 85.1% of molecular functions, 76.9% of biological processes, and 74.6% of cellular locations can be correctly assigned(Roy et al., 2010).

The model of LSP-1 has a structural conservation with spectrin (TM-score = 0.678) and actin (TM-score = 0.556). The protein has functional annotations in actin filament organization (GO-score = 0.36) and cell connection (GO-score = 0.36). It could have a H^+/K^+-exchanging ATPase or xenobiotic transporter activity according to the bioinformatic analysis. However, the confidence score ($Cscore^{EC}$ = 0.117) was not high.

The structural analog analysis in the modelling shows NCU02540 protein has substantial conservation (TM-score = 0.625) with the yeast Lsp1 protein which is an eisosomal protein but is not functional in eisosome formation (Walther et al., 2006). It was predicted as an integral cell membrane component ($Cscores^{GO}$ = 0.16, GO-score = 0.36). It has annotations of functions of Na^+/K^+ transferase activity and of ATP synthesis based on the protein modelling analysis ($Cscore^{EC}$ = 0.117).

For protein NCU03647, six conserved protein superfamilies were detected, some of which are the eisosome-1 superfamily required for normal formation of eisosomes; the SMC_prok_A superfamily playing roles in organizing and segregating chromosomes for partition; the Pneumo_att_G superfamily of attachment membrane glycoprotein G. The NCU03647 protein has structural similarity with human ATR-ATRIP complex (TM-score = 0.994). The silico analysis indicates it has endo-alpha-N-acetylgalactosaminidase activity ($Cscore^{EC}$ = 0.133) and is associated with protein binding ($Cscores^{GO}$ = 0.22, GO-score = 0.58).

NCU07366 protein has conserved domains belonging to five superfamilies: the PLN02981 superfamily is describe as the glucosamine:fructose-6-phosphate aminotransferase; the GlmS superfamily is the glucosamine-6-phosphate (GlcN6P) synthetase containing amidotransferase and phosphosugar isomerase domains; the glmS superfamily including the glucosamine-fructose-6-phosphate aminotransferase (isomerizing); the GFAT superfamily that is found at the N-terminus of GlcN6P synthase, the domain catalyzes amide nitrogen transfer from glutamine to the appropriate substrate; and the SIS superfamily including the sugar Isomerase that is found in many phosphosugar isomerases and phosphosugar binding proteins. The structure alignment of the model shows that NCU07366 has a similarity with the GlcN6P synthase (TM-score = 0.85). In agreement with the analog result, it is confidently verified that NCU07366 has the annotation of GlcN6P synthase activity ($Cscore^{EC}$ = 0.61). In addition, the protein has an annotation of carbohydrate binding ($Cscores^{GO}$ = 0.58, GO-score = 0.83) and has an annotation of binding function that interacts with proteins or protein complexes ($Cscores^{GO}$ = 0.58, GO-score = 0.67), which well explains the colocalization of NCU07366 and eisosomes.

NCU02425 has conserved domains belonging to PH (Pleckstrin Homology) and PH-like superfamily. These superfamilies generally play roles in targeting proteins to the appropriate cellular location or in the interaction with a binding partner and are found in cytoskeletal

associated molecules as well as in eukaryotic signaling proteins. The model of NCU02425 protein is similar to RNA binding protein (the Cas13b binary complex) at the structural level (TM-score = 0.90). The protein modelling analysis was not able to assign any enzyme activity with confidence but found that it has annotations of protein binding function ($Cscores^{GO}$ = 0.17, GO-score = 0.39) as well as function in the location establishment of cellular components ($Cscores^{GO}$ = 0.17, GO-score = 0.33). What is more, it is predicted to be involved in protein-containing complexes localized at the cell periphery (GO-score = 0.33) and to have roles in cellular protein-containing complex assembly (GO-score = 0.33). The analysis indicated that the protein most probably establishes the localization and formation of eisosomes.

A MARVEL superfamily conserved domain was detected during the analysis of protein NCU04639. It is a membrane-associating domain usually found in lipid-associating proteins and may play roles in membrane apposition and vesicle transport. The model of NCU04639 has structural analogs such as oxidoreductase (Cytochrome b561, TM-score = 0.80; Human duodenal cytochrome b, TM-score = 0.77), lipid G protein coupled receptor (TM-score = 0.63) and the cytochrome C family protein (TM-score = 0.63). The functional predictions showed that it is an integral component of the membrane ($Cscores^{GO}$ = 0.22, GO-score = 0.53) that plays roles in the G protein-coupled receptor signaling pathway ($Cscores^{GO}$ = 0.22, GO-score = 0.53) and defense response ($Cscores^{GO}$ = 0.22, GO-score = 0.35). Furthermore, it has annotations associated with the glycosaminoglycan catabolic process (GO-score = 0.44) and cell wall macromolecule metabolic process (GO-score = 0.44).

Similar to the NCU04639 protein, NCU05230 also contains a conserved domain that belongs to the MARVEL superfamily and has the same structural analogs (oxidoreductases, TM-scores = 0.84, 0.83; lipid G protein coupled receptor, TM-score = 0.61), which reveals that these two proteins probably have similar function(s) at the plasma membrane. NCU05230 is predicted to relate to the G protein-coupled receptor signaling pathway ($Cscores^{GO}$ = 0.15, GO-score = 0.47) and establishment of cellular component localization (GO-score = 0.32). In addition, it has a transmembrane signaling receptor functional annotation (GO-score = 0.56) and is predicted to take part in the phosphate-containing compound metabolic process (GO-score = 0.56), light stimulus detection process (GO-score = 0.56) and cellular protein modification process (GO-score = 0.35). It is not only predicted as an integral component (GO-score = 0.56), but also as a photoreceptor inner/outer segment at the plasma membrane (GO-score = 0.56).

According to the structural and functional prediction results of each MCC/eisosomal core component, NCU02425 establishes the location and promotes the assembly of eisosomes and then is bound with LSP-1 (NCU07495); LSP-1 and NCU02540 grasp the cell membrane and form the furrow structures of eisosomes; NCU03647 and NCU07366 bind to LSP-1 at eisosomes on the cytoplasmic side of the cell membrane and carry out their respective functions; NCU04639 and NCU05230 are transmembrane proteins localized at the furrow of MCC and may have connections with NCU02540 because the later has the Na^+/K^+ transferase activity, which needs transmembrane protein domains (**Figure 4**).

References

Roy, A., Kucukural, A., and Zhang, Y. (2010). I-TASSER: a unified platform for automated protein structure and function prediction. 5, 725–738. doi:10.1038/nprot.2010.5.I-TASSER.

Walther, T. C., Brickner, J. H., Aguilar, P. S., Bernales, S., and Walter, P. (2006). Eisosomes mark static sites of endocytosis. *Nature* 439, 998–1003. doi:Doi 10.1038/Nature04472.

Supplementary Table S1. genes and their productions in LC-MS result

gene ID (from FungiDB)	gene production
NCU07495	sphingolipid long chain base-responsive protein LSP1
NCU03647	hypothetical protein
NCU02540	meiotic expression up-regulated protein 14
NCU02120	hypothetical protein
NCU02425	PH domain-containing protein
NCU05803	translational activator
NCU07366	glutamine-fructose-6-phosphate aminotransferase
NCU03897	RNA binding effector protein Scp160
NCU06943	SIK1
NCU02839	T-complex protein 1
NCU06544	protein kinase C-like protein
NCU09808	dynamin-1
NCU08875	Cullin binding protein CanA
NCU08699	bli-4 protein
NCU02207	T-complex protein 1 subunit beta
NCU05887	oxidoreductase
NCU09700	T-complex protein 1 subunit beta
NCU08340	ADP-ribosylation factor 1
NCU03102	40S ribosomal protein S11
NCU00410	eukaryotic release factor 1
NCU07567	T-complex protein 1 subunit theta
NCU09119	ATP synthase subunit gamma
NCU08920	ATP-binding cassette sub-family F member 2

Supplementary Table S2. strains used in our research

Strain	Genotype	Source
FGSC6103	*his-3* (Y234M723) mat A	Fungal Genetics Stock Center
FGSC9716	*his-3* (Y234M723) mat a	Fungal Genetics Stock Center
QY127-4	*NCU04809::gfp*	This study
NcT462	*lsp-1::rfp* mat a	This study
NcT475	*lsp-1::rfp* mat A	This study
NcT463	*NCU06384::gfp*	This study
NcT464	*NCU03571::gfp*	This study
NcT465	*NCU07334::gfp*	This study
NcT466	*NCU01065::gfp*	This study
NcT467	*lsp-1::gfp*	This study
NcT468	*NCU04195::gfp*	This study
NcT469	*NCU06352::gfp*	This study
NcT470	*NCU05198::gfp*	This study
NcT471	*NCU00586::gfp*	This study
NcT474	*NCU07754::gfp*	This study
NcT510	*NCU05230::gfp*	This study
NcT511	*NCU04639::gfp*	This study
NcT512	*NCU02540::gfp*	This study
NcT515	*NCU07754::gfp*	This study
NcT516	*NCU03647::gfp*	This study
NcT517	*NCU02425::gfp*	This study
NcT518	*NCU07366::gfp*	This study

Supplementary Table S3. plasmids used in the study

plasmid	inserted fragment
pAS827	*NCU05198::gfp*
pMF844	*NCU00586::gfp*
pAA846	*NCU07495::gfp*
pCE847	*NCU07754::gfp*
pBS848	*NCU06384::gfp*
pQY849	*NCU06352::gfp*
pQY853	*NCU07334::gfp*
pQY854	*NCU03571::gfp*
pQY855	*NCU04809::gfp*
pQY856	*NCU01065::gfp*
pQY857	*NCU04195::gfp*
pQY882	*NCU03647::gfp*
pQY883	*NCU07366::gfp*
pQY884	*NCU02425::gfp*
pQY885	*NCU05230::gfp*
pQY887	*NCU04639::gfp*
pQY888	*NCU02540::gfp*
pQY860	*N. crassa lsp-1 promoter*
pQY867	*lsp-1 promoter::NCU07495::gfp*
pQY868	*lsp-1 promoter::NCU07495::rfp*

Supplementary Table S4. primers used for the amplification of aim genes in our study

primer	sequence	combined fragment
FK2654	5'- GGAGACAUGGCGCGCCATGTTGTCAACCGGAGTGGAC -3'	*NCU05198*
FK2655	5'- ACAAGCCUAAAAAGCCAATCCAAAACCCATTCGAG -3'	*NCU05198*
MF2867	5'- GGAGACAUATGAACAAATCTCGTAAGTTGAT -3'	*NCU00586*
MF2868	5'- ACAAGCCUGTAGTCATCCTTGACCCT -3'	*NCU00586*
QY3213	5'- CGGGATCCCGATGCATCGAACCTACTCCATGCG -3'	*NCU07495*
QY3214	5'- CGGAATTCCTTGTACAGCTCGTCCATGCC -3'	*NCU07495*
JG2838	5'- GGAGACAUATGGGCACCGACACAAAAGGG -3'	*NCU07754*
JG2839	5'- ACAAGCCUGTGTATAGCTCCGTAACTGCCG -3'	*NCU07754*
JG2832	5'- GGAGACAUATGGGTTACACCACTCTCTGG -3'	*NCU06384*
JG2833	5'- ACAAGCCUAACACGCTCGCTATGAAAAGACTG -3'	*NCU06384*
QY2890	5'- GGAGACAUATGACGCCTGACCGAGATAGG -3'	*NCU06352*
QY2891	5'- ACAAGCCUCCAAACATCAGGCCCAAACCTTC -3'	*NCU06352*
QY2898	5'- GGAGACAUATGGCAGAGAAACTCAGCGCC -3'	*NCU07334*
QY2899	5'- ACAAGCCUGACAATCTTGCTGTCATCTGCACC -3'	*NCU07334*
QY3069	5'- TTGGCGCGCCATGAACGGAGACTTCAGCCTGT -3'	*NCU03571*
QY3070	5'- CCCAAGCTTAAAAGCAGCCCCCAACCCAT -3'	*NCU03571*
QY2914	5'- GGAGACAUATGTTCGGATTCGGCCAGCG -3'	*NCU04809*
QY2915	5'- ACAAGCCUCTGAGCAGGACTTACCTTCTTC -3'	*NCU04809*
QY2916	5'- GGAGACAUATGTCGTCCGGCCCCGTTGAA -3'	*NCU01065*
QY2917	5' ACAAGCCUGACCTGCTTCTCAGGGTCCCT --3'	*NCU01065*
QY2912	5'- GGAGACAUATGGTGACCCAAAGTGACGAC -3'	*NCU04195*
QY2913	5'- ACAAGCCUTCGCCCACTAATCTTCTTCTCC -3'	*NCU04195*

QY3203	5'- AAAAAGCAGGCTATGGTCTCATCTACTGCGCTTACG -3'	*NCU03647*
QY3204	5'- AGAAAGCTGGGTATCCTCCTTATCAAAATTCTCCG -3'	*NCU03647*
QY3209	5'- AAAAAGCAGGCTATGTGGTACGTTAAAGAGAACCT -3'	*NCU07366*
QY3210	5'- AGAAAGCTGGGTCTCCACAGTCACCGACTTG -3'	*NCU07366*
QY3201	5'- AAAAAGCAGGCTATGATGACCGCTAGATCTCC -3'	*NCU02425*
QY3202	5'- AGAAAGCTGGGTCACTAAGCGGGGAAACGG -3'	*NCU02425*
QY3034	5'- GGAGACAUATGCTCGCCGGTTTATGTATC -3'	*NCU05230*
QY3035	5'-ACAAGCCUAACATACGACCTCGACCCCA-3'	*NCU05230*
QY3036	5'- GGAGACAUATGGAAGTGATACCCCTCATC -3'	*NCU04639*
QY3037	5'- ACAAGCCUGACGACCACCTGTGACAT -3'	*NCU04639*
QY3032	5'- GGAGACAUATGTGAGTTAGCCCACATGTAATC -3'	*NCU02540*
QY3033	5'- ACAAGCCUAACAGCAGTTGTCTGCACC -3'	*NCU02540*
QY2942	5'- CCTTAATTAAGGTGAGGACGGATGAATAAGAC -3'	*N. crassa lsp-1 promoter*
QY2943	5'-TTGGCGCGCCGGTGGATTAGGTAGTATTTATCGATTG-3'	*N. crassa lsp-1 promoter*

Supplementary figures

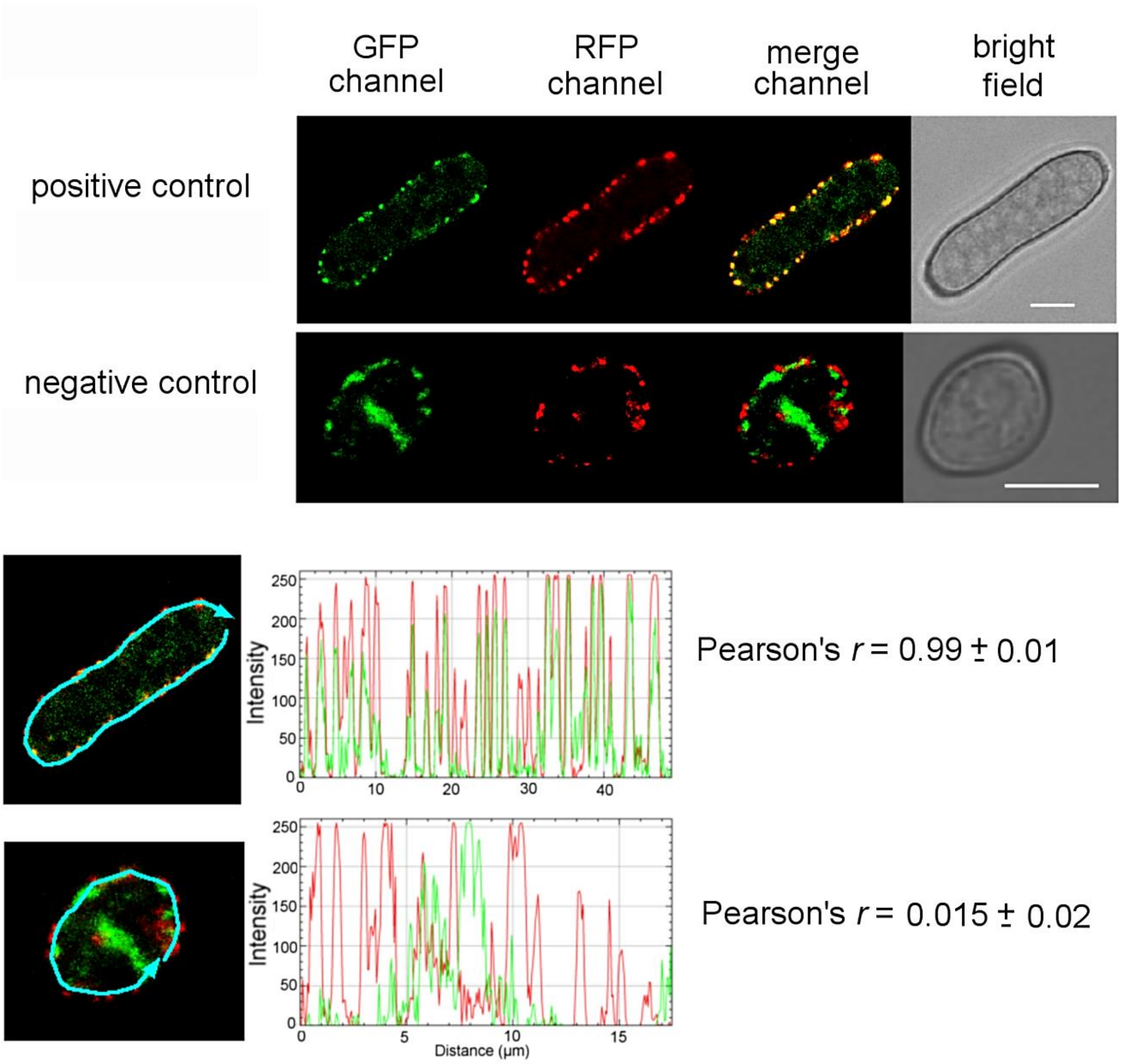

Supplementary Figure 1. The positive control versus the negative control for the colocalization analysis with LSP-1::RFP. Positive control: LSP-1::GFP; negative control: NCU03571::GFP. Arrow lines show the routes of the fluorescence density profile analysis. Scale bar = 5 μm.

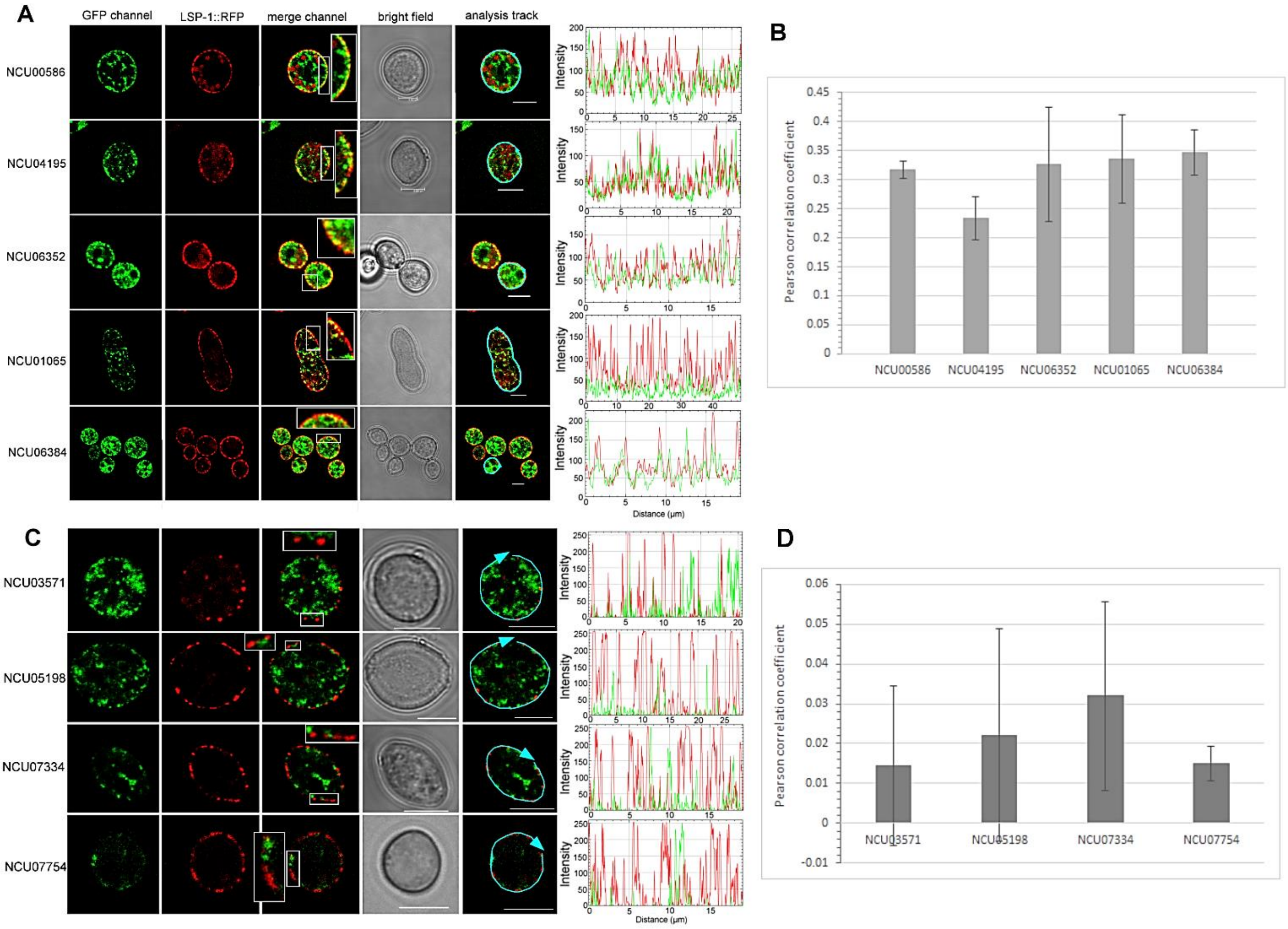

Supplementary Figure 2. Localization verification of the eisosome associated proteins and non-eisosome proteins identified in our study. A. The dual fluorescence colocalization analysis of LSP-1::RFP and eisosome associated proteins tagged by GFP. **B.** The Pearson correlation coefficients on the colocalization between the eisosome associated proteins and eisosomes. **C.** The colocalization analysis of LSP-1::RFP and non-eisosome proteins tagged by GFP. **D.** The Pearson correlation coefficients on the colocalization between non-eisosome proteins and eisosomes. Arrow lines show the routes of the fluorescence density profile analysis, boxes show magnified cell peripheries with more details. Scale bar = 5 μm.

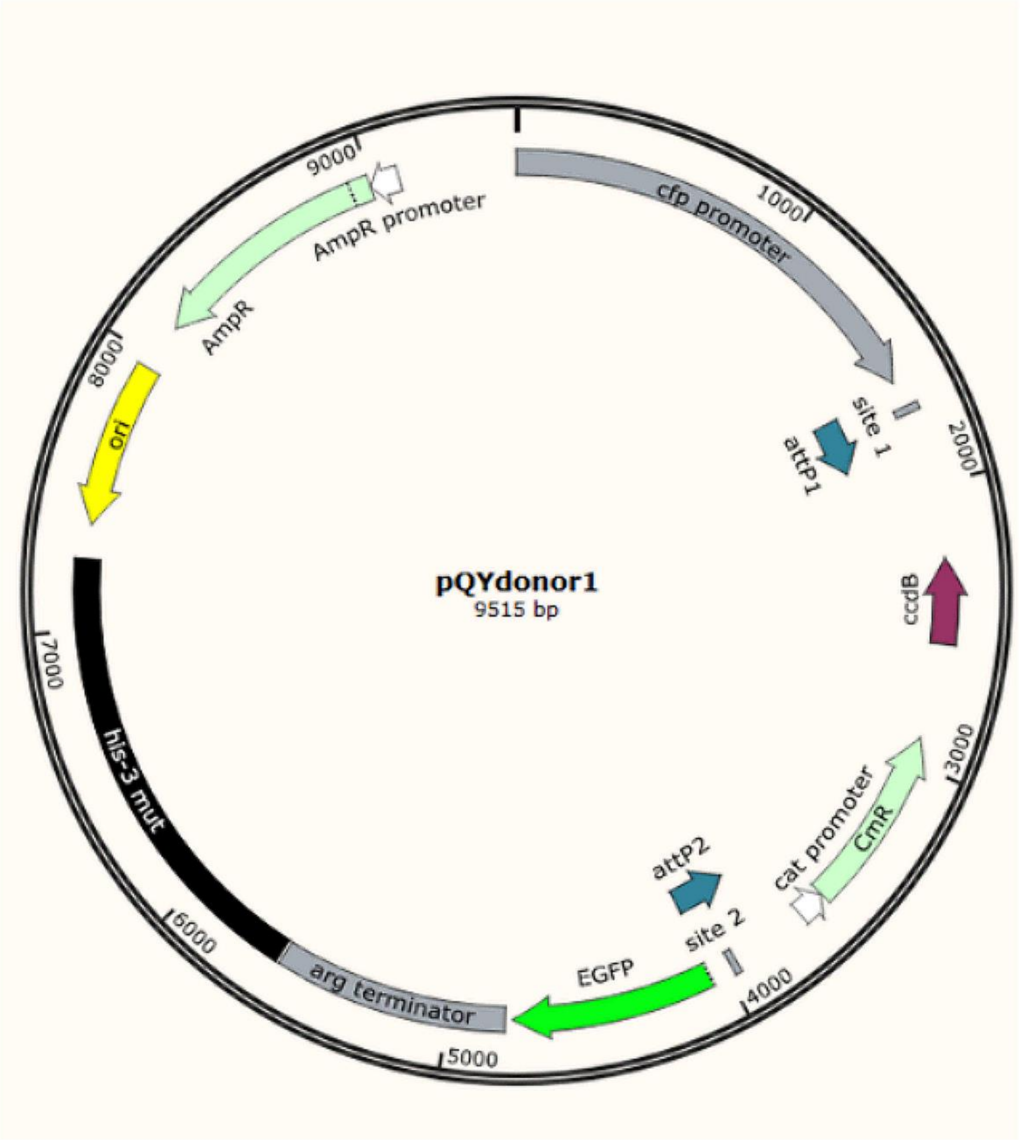

Supplementary Figure 3. The *N. crassa* expression vector used in the research.
